# Brazilian dance self-perceived impacts on quality of life of people with Parkinson’s

**DOI:** 10.3389/fpsyg.2024.1356553

**Published:** 2024-02-21

**Authors:** Marcela dos Santos Delabary, Isadora Loch Sbeghen, Eliamary Cristiane Teixeira da Silva, Carlos Cristiano Espedito Guzzo Júnior, Aline Nogueira Haas

**Affiliations:** ^1^Department of Physical Education, Physiotherapy and Dance, Federal University of Rio Grande do Sul, Rio Grande do Sul, Brazil; ^2^Atlantic Fellow for Equity in Brain, Global Brain Health Institute, Trinity College Dublin, Dublin, Ireland

**Keywords:** parkinsonian disorders, therapy through dance, quality of life, social isolation, COVID-19

## Abstract

**Background:**

Parkinson’s disease (PD) causes several motor and non-motor symptoms, resulting in negative impacts on physical, mental, emotional, and social aspects of people with PD quality of life. Dance has been considered as a potential non-pharmacological intervention to improve people with PD motor and non-motor symptoms, thereby enhancing quality of life.

**Purpose:**

To analyze the self-perceive impacts of Brazilian Dance on the quality of life (physical, mental, emotional, and social) of PwPD, both before and during the COVID-19 pandemic.

**Methods:**

Fourteen participants from the “Dança & Parkinson” project were included in this qualitative study. Data collection instruments consisted of a profile and personal data sheet; assessment of accessibility to the online dance classes; Telephone Montreal Cognitive Assessment by phone call; and semi-structured interview conducted through ZOOM video call. The participants characterization data were calculated using mean, standard deviation, and percentages with the Excel Program version 2013. Qualitative data was analyzed using the Thematic Analysis technique in the Nvivo, version 8.0, qualitative analysis of text, sound, and video program.

**Results:**

The participants reported facing various challenges in dealing with PD, which negatively impact their quality of life. However, their resilience, acceptance, and dedication to treatment play an important role in coping with the issues related to the disease. Brazilian dance, both in-person before the COVID-19 pandemic and online during the pandemic, led the participants to perceive improvements in physical, mental, emotional, and social aspects of quality of life.

**Conclusion:**

The Brazilian dance appears to have a positive impact on the physical, mental, emotional, and social aspects of the participants’ quality of life, both before and during the COVID-19 pandemic.

## 1 Introduction

Parkinson’s disease (PD), a neurodegenerative, chronic, progressive, and multifactorial condition, is currently the fastest-growing neurological disorder worldwide (Poewe et al., 2017; Simon et al., 2020). People with PD (PwPD) experience motor and non-motor symptoms, caused by the death of dopaminergic neurons in the substantia nigra of the basal ganglia (Poewe et al., 2017; Balestrino and Schapira, 2020). As PD progresses, PwPD face diminishing functional independence, reduced well-being and self-esteem, difficulties in performing daily activities, and limitations in social participation, all of which negatively impact their quality of life (QoL) (Martinez-Martin, 2017; Valcarenghi et al., 2018; Vescovelli et al., 2018; Kuhlman et al., 2019; Verity et al., 2020).

Engaging in complementary non-pharmacological interventions is important for preserving motor and cognitive skills, and helps minimize the impact of PD on QoL (Martinez-Martin, 2017; Donley et al., 2019). Several studies have shown that dance is an accessible non-pharmacological intervention (Emmanouilidis et al., 2021) that promotes various motor and non-motor benefits, for instance, improvements in gait and functional mobility (Delabary et al., 2020), as well as in cognitive domains such as executive function (Kalyani et al., 2019), contributing to improved QoL in PwPD (Hackney and Bennett, 2014; Holmes and Hackney, 2017). However, there is no consensus in the literature regarding the effects of dance on PwPD QoL, indicating varying or inconclusive results. Some systematic reviews show significant improvements in overall QoL of PwPD (Shanahan et al., 2015; Carapellotti et al., 2020), while others do not (Delabary et al., 2018; Ismail et al., 2021; Zhou et al., 2021).

Quality of life is a comprehensive concept influenced by an individual’s physical health, psychological state, level of independence, social relationships, and their connection to significant aspects of their environment (WHOQOL Group, 1993; Stocchi et al., 2014). Therefore, any assessment of QoL in PwPD should be subjective, individualized, multidimensional, and consider the person’s self-perceived social, psychological and physical condition in relation to the disease (Martinez-Martin, 2017). Since QoL is a multifaceted concept with a unique meaning for each individual, relying solely on quantitative assessments can inhibit a thorough understanding of this concept, this being a limitation in several studies on the effects of dance on the QoL of PwPD (Delabary et al., 2018; Ismail et al., 2021; Zhou et al., 2021). Nevertheless, studies using a qualitative approach to analyze the impact of dance on the QoL of PwPD have demonstrated improvements in: social participation (Zafar et al., 2017); self-confidence and participation in activities of daily living (Holmes and Hackney, 2017); body control and awareness, and motor symptoms (Beerenbrock et al., 2020); and the achievement of new ways of moving through experimentation and adaptation (Hulbert et al., 2020). However, according to the authors’ knowledge, no studies has analyzed the self-perceived impacts of Brazilian Dances on the QoL of PwPD, using a qualitative approach.

In 2020, the decline in physical and social activities due to the COVID-19 pandemic had a significant detrimental effect on the PwPD QoL (Subramanian et al., 2020). This was shown in deterioration of motor symptoms, such as increased rigidity, tremors, gait difficulties, and non-motor symptoms (Brown et al., 2020; Subramanian et al., 2020), including mood swings, cognitive issues, fatigue, anxiety, depression, and fear of death (Brown et al., 2020; Haas et al., 2022; Moratelli et al., 2022). Online non-pharmacological interventions (Shalash et al., 2021), including dance classes, emerged as important tools to mitigate the negative effects of the pandemic on this population (Bek et al., 2021; Morris et al., 2021; Delabary et al., 2022; Walton et al., 2022).

Therefore, gaps in the current literature center around the need for more studies employing qualitative methodological approach, conducting a comprehensive exploration about the various aspects of PwPD QoL influenced by different dance genres, and to understand the impacts caused by the COVID-19 pandemic. Thus, this study aims to analyze the self-perceive impacts of Brazilian Dance on the quality of life (physical, mental, emotional, and social) of PwPD, both before and during the COVID-19 pandemic.

## 2 Materials and methods

### 2.1 Study design

This is a qualitative study approved by the Ethical Committee of the School of Physical Education, Physical Therapy and Dance at the Federal University of Rio Grande do Sul (CAAE 33547920.9.0000.5347). All the participants signed a consent form, and the study followed the Consolidated criteria for reporting qualitative studies (COREQ) and Standards for Reporting Qualitative Research (SRQR).

### 2.2 Participants

The participants were: people with a clinical confirmed diagnosis of PD, according to the London Brain Bank Criteria (National Collaborating Centre for Chronic Conditions, 2006), undergoing medical treatment for PD for at least 1 year, with regular use of anti-parkinsonian drugs; both sexes; ≥50 years old; staging between 1 and 3 of the Hoehn and Yahr Scale (H&Y), able to walk independently; who participated in the “Dance & Parkinson” project of the Federal University of Rio Grande do Sul before the COVID-19 pandemic, and continued to participate in the “Dance & Parkinson at home” online project, during the pandemic. Those with risk factors, such as recent surgery, deep brain stimulation, and other associated neurological or chronic diseases were excluded.

All the participants in the “Dance & Parkinson’s” project who, during the pandemic, took part in the online classes were invited to participate in the study. They were invited to participate in the study via WhatsApp, and all accepted. Thus, 14 PwPD (9 women and 5 men) were enrolled in the study and assigned names of colors to maintain their anonymity. The participants’ demographic and clinical characteristics are presented in Table 1.

**Table 1:** Participants’ demographic characteristics.

Participant	Duration of interview	Sex	Declared ethnicity	Age (years)	Time of PD (years)	Time of dance practice (years)	H&Y	Cognition (T-MoCa)	Level of education (years)	Medication LED (mg/day)
Lilac	40 min	F	White	62	7	6	2	19	Higher Education	Prolopa 400
Turquoise	72 min	F	White	56	9	2	3	22	Higher Education	Prolopa 800
Yellow	33 min	F	White	76	4	4	3	16	No level	Prolopa 500
Orange	42 min	F	White	74	6	4	2.5	18	High School	Prolopa 300
Red	62 min	F	White	87	8	6	3	15	No level	Prolopa 400
Navy Blue	47 min	M	White	67	2	2	2	22	High School	Prolopa 600
Mustard	42 min	M	White	70	5	3	1	21	Higher Education	Prolopa 200
Light Green	30 min	F	Black	67	3	3	3	15	No level	Prolopa 300
Brown	29 min	M	White	67	3	2	2.5	16	High School	Prolopa 400
Violet	32 min	F	White	85	24	3	1.5	21	High School	Prolopa 800
Burgundy	40 min	F	Black	68	14	6	3	18	High School	Cardiodopa 1,200
Dark Green	46 min	F	White	66	6	3	3	15	No level	Prolopa 800
Pink	38 min	F	White	67	6	3	3	19	High School	Prolopa 800
Gray	42 min	M	White	83	6	3	3	16	Elementary School	Prolopa 300
Mean	42.5	NA	NA	71.1	7.4	3.6	2.6	18.1	NA	607.1
SD	11.9	NA	NA	8.9	5.7	1.5	0.7	2.6	NA	239.9
%	NA	64% F 36% M	86% W 14% B	NA	NA	NA	NA	NA	21% HE 43% HS 7% ES 29% NL	7% LC 93% P

Parkinson’s disease (PD); minutes (min); Hoehn & Yahr (H&Y) scale; Telephone Montreal Cognitive Assessment Test (T-MoCA); Standard deviation (SD); Not applicable (NA); Female (F); Male (M); White (W); Black (B); Higher Education (HE); High School (HS); Elementary School (ES); No level (NL); Levodopa-Carbidopa (LB); Prolopa (P). Levodopa equivalent daily dose (LED) (Tomlinson et al., 2010).

### 2.3 “Dance & Parkinson’s” project

**Figure 1:**
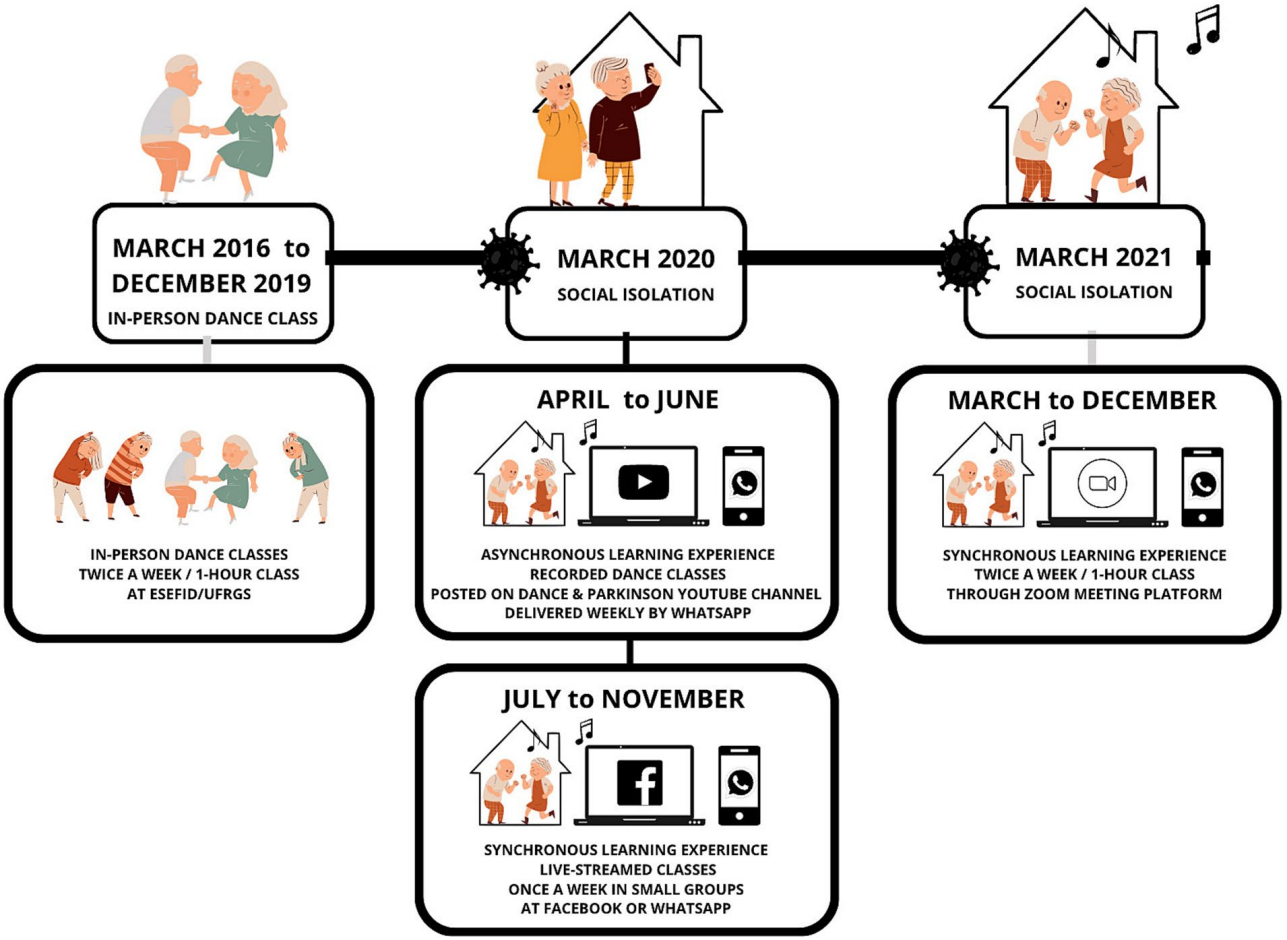
“Dance & Parkinson’s” project timeline form March 2016 to December 2021.

The community and research project “Dance & Parkinson’s” for the School of Physical Education, Physiotherapy and Dance (ESEFID) at the Federal University of Rio Grande do Sul (UFRGS), Brazil, was created in 2016. The project is led by an Associate Professor in Dance and organized by a group of undergraduate and post-graduate students. Since 2016, the “Dance & Parkinson’s” project has promoted dance classes, inspired by the Brazilian rhythms (Haas et al., 2018; Peyré-Tartaruga et al., 2022), designed to provide qualitative improvements in the physical and mental health of PwPD. Figure 1 presents the project timeline from March 2016 to December 2021.

From March 2016 to December 2019, the Brazilian dance classes were performed in person, 1-h twice a week at the ESEFID. In March 2020, the COVID-19 pandemic was officially declared in Brazil, social isolation was adopted as a preventive measure, and the project was adapted for the online format. The participants attended online dance classes, with asynchronous and synchronous learning experience (58). From April to July 2020, the project offered recorded dance classes on a dedicated Youtube Channel.^1^ During the second half of 2020, a synchronous learning experience with live-streamed, weekly dance classes, involving small groups was offered via WhatsApp Video Call or Facebook Messenger Room (58). In March 2021, the project offered synchronous learning experience with 1-h online dance classes twice a week through ZOOM platform.

**Figure 2:**
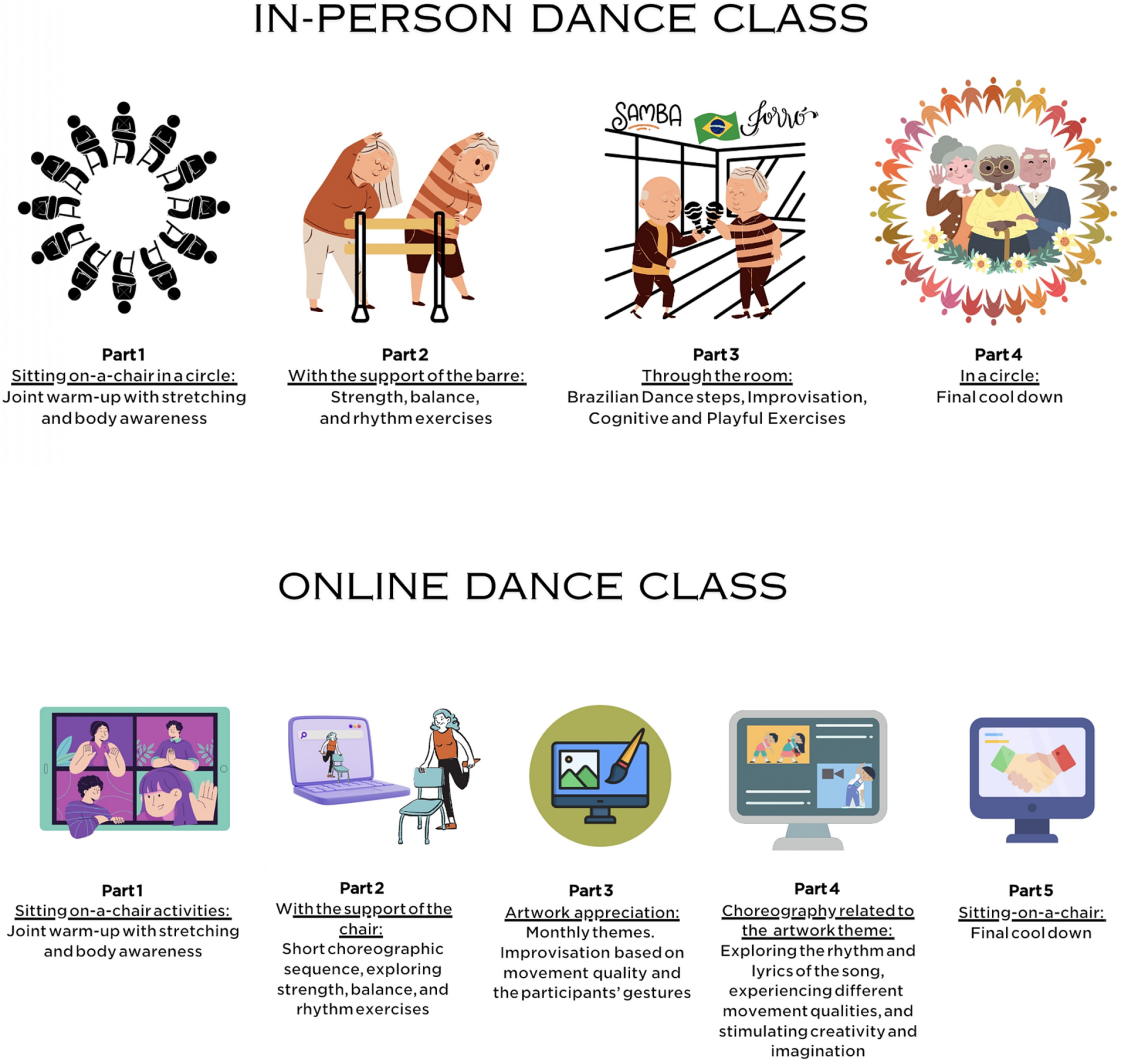
Structure of the in-person and online Brazilian dance classes.

The in-person and online Brazilian dance classes were structured according to Haas et al. (2018) and Delabary et al. (2022) as shown in the Figure 2.

### 2.4 Instruments

Before interviewing, during an initial phone call, anamnesis was carried out to characterize each participant’s demographic characteristics, and the Telephone Montreal Cognitive Assessment (T-MoCA) were applied to assess the participant’s cognitive level (Pendlebury et al., 2013). In addition, each participant was asked to send a certificate from their neurologist with the Hoehn & Yahr scale (H&Y) staging.

A semi-structured interview with open-ended questions was developed based on a literature review regarding the PD characteristics and impacts and the benefits of dance practice on PwPD QoL. At the beginning and the end of the semi-structured interview were asked. The main body of the interview focused on four primary categories: (1) Living with Parkinson’s; (2) Self-perceived QoL; (3) Self-perceived impacts of the in-person Brazilian dance classes; and (4) Self-perceived impacts of the online Brazilian dance classes (Table 2). Within these primary categories, three main subcategories were created: (1) Physical; (2) Mental and emotional; and (3) Social.

**Table 2:** Semi-structured interview questions.

Topic/categories	Questions
Beginning	- Tell me a little about your experience in the “Dance & Parkinson” project. Why did you join the project? What were your expectations before joining? And what are your expectations now with the online dance classes? - Tell me a little about your daily routine before the pandemic and now during the pandemic.
Self-perceived QoL (physical, mental/emotional and social aspects)	- What is QoL for you? - Do you perceive any impact of PD: on your activities of daily living (ADLs) and functional independence? On your self-esteem? On your sense of well-being? On your social life prior to or during the COVID-19 pandemic?
Self-perceived impacts of the in-person Brazilian dance classes (physical, mental/emotional, and social aspects)	- Do you perceive any impact of in-person dance classes: on your ADLs and functional independence? On your self-esteem? On your sense of well-being? On your social life prior to the pandemic?
Self-perceived impacts of the online Brazilian dance classes (physical, mental/emotional, and social aspects)	- And now, during the pandemic, do you perceive any impact of online dance classes: on your ADLs and functional independence? On your self-esteem? On your sense of well-being? On your social life?
End	- Do you have any other information that you want to share? Any other questions that I have not asked, that you want to talk about?

The interviews were conducted on the ZOOM platform by a dance teacher and researcher, and lasted 43 ± 12 min on average. All the semi-structured interviews took place during the “ON” state of the anti-parkinsonian medications, up to 2 h after taking the medicine.

### 2.5 Data analysis

The semi-structured interviews were conducted and recorded on the ZOOM platform. Later, they were transcribed faithfully by dance researchers, preserving the original words and reviewed by the participants. The content of the interviews was then analyzed to extract meaning and gain insight into the collected data to address the research questions.

The Qualitative Text, Sound, and Video Analysis program, Nvivo version 1.5.1, was used to organize, code, store, and analyze the qualitative content. In the data analysis, we implemented a detailed coding process to systematically analyze text data, ensuring a comprehensive exploration of the participants’ self-perceived impacts of Brazilian Dance on their QoL. To enhance the reliability of the data analysis, an analyst independently coded a subset of the data, aiming to mitigate potential errors and strengthen the robustness of our findings and ensuring the reliability of Nvivo software by emphasizing proper coding practices, mitigating errors, or misinterpretation risks in the qualitative content analysis. During the coding process, the interviews were read and analyzed, using the criteria of the Thematic Analysis method (Braun and Clarke, 2006), and, as we previously mentioned, four primary categories were identified (Live with Parkinson’s; Self-perceived QoL; Self-perceived impacts of the in-person Brazilian dance classes; and Self-perceived impacts of the online Brazilian dance classes). The results are organized according to these four primary categories using interview excerpts, while subcategories are grouped in tables, and subdivided into subthemes, considering physical, mental and emotional, and social aspects of QoL.

The qualitative analysis was conducted in the original language, Brazilian Portuguese. To present these results, relevant excerpts from the interviews were translated to English. Back-translation process, involving a bilingual researcher and a proof-reader English speaker, was used to enhance the accuracy of translation and minimizing the risk of errors or misinterpretation.

Statistical analysis (mean, standard deviation, and percentage) are used to present the participants’ demographic characteristics (Table 1). The analysis was carried out using the Excel Program, version 2013.

## 3 Results

### 3.1 Living with Parkinson’s

The participants shared their experiences, highlighting the difficulties they faced upon receiving a PD diagnosis and the subsequent challenges in adapting their daily lives and routine. Brown expressed the inevitability of PD having a significant impact: *“There’s no way something like this could pass by without having an impact, right?.”* Yellow described the hardships of living with Parkinson’s, affecting communication and mobility: *“It’s bad, it’s hard to have Parkinson’s. So, it’s hard to talk, to move, people have to be patient to listen to you […]. Everything became more difficult, communication, movements. Everything.”* Burgundy emphasized the lifestyle changes and the constant reminder of the disease: *“This changes your rhythm of life, your routine, because you are always thinking about the medicine, even if you do not want to think about the disease, you have to remember to take the medicine on time, you have to take care of your diet with the medicine […] you have to know how to let go, because otherwise you get paranoid. That’s the truth.”*

Some participants also mentioned the sense of losing their essence and not feeling like themselves anymore. Turquoise reflected on this loss of identity: *“I think it is one of the worst feelings because you come to believe that you are no longer yourself.”* Burgundy expressed the complications of the disease, leading to a sense of not being the same person anymore: *“It’s complicated, right? It’s depressing because you are no longer the same person, right? It’s not 100 % your way of being… walking, doing this or that …”*

Many participants highlighted the importance of accepting the reality of Parkinson’s as a part of life. They emphasized the need to confront the disease head-on rather than dwelling on self-pity. For example, Lilac stated: *“As soon as Parkinson’s appeared, I was left with no direction… But then you get over it, you take it as a normal thing… There’s no point in me crying in the corner because I have Parkinson’s.”* Turquoise added: *“You learn to try to get around it and see the other side! […] taking it in a sporting way or it’s too sad!.”*

The participants stressed the significance of staying positive in the face of adversity. They refused to surrender to the disease and maintained an optimistic mindset: *“We cannot surrender, that’s what I always think, the disease wants to take me down but I do not let it, so I think positively.” (Burgundy); “I do not give up, I may not be very good, but I do not give up!”* (*Pink*). Some participants found solace in accepting their condition as part of a higher plan, citing their faith in God: *“I accept things as they are… I think that things have to be this way, and we have to accept it, it is God’s will, so, I am catholic, and I accept everything.” (Yellow); “I am not ashamed of my tremor, I am not ashamed of having the disease because God gave it to me and I have to put up with it, right?” (Burgundy).*

For certain participants, a pragmatic approach involved acknowledging that certain things are beyond their control. They chose to accept their situation and adapt to it: *“I’m a very realistic person, and I accept things […] I accepted my Parkinson’s well.” (Violet); “If it has to be, it has to be, and that’s it. Just accept it and get used to it.” (Mustard).*

The participants also recognized the importance of actively seeking ways to improve their quality of life. They assumed responsibility for their well-being and treatment, striving to make the most of their situation: *“So, I have to try to do my best, look for alternatives to improve my quality of life. […] While there is no cure, we have to live with it and try to do the best we can.” (Lilac); “I have Parkinson’s, and I will do my best to live as well as I can and not to give my children and family too much trouble.” (Orange).*

The participants highlighted various physical, mental, emotional, and social challenges in their daily lives, all stemming from the limitations imposed by PD. These challenges had a profound impact on their social interactions and QoL. Despite experiencing the physical, mental, emotional, and social impacts of PD, resilience and acceptance were common themes among nearly all the participants. These two qualities appeared to be essential for coping with the challenges posed by the disease and the necessary adjustments in their lives. The self-perceived impacts of living with Parkinson’s, categorized into physical, mental and emotional, and social aspects are summarized in Table 3.

**Table 3:** The self-perceived impacts of living with Parkinson’s.

Subcategories of the impacts of living with Parkinson’s		Lilac	Turquoise	Yellow	Orange	Red	Navy Blue	Mustard	Light Green	Brown	Violet	Burgundy	Dark Green	Pink	Gray	*n*	%
Physical	Slowness	**x**		**x**			**x**	**x**	**x**		**x**			**x**		7	50
	**Rigidity**	**x**		**x**	**x**	**x**	**x**	**x**		**x**		**x**	**x**	**x**		**10**	**71**
	Tremor	**x**				**x**	**x**				**x**	**x**			**x**	6	43
	Balance Changes					**x**		**x**	**x**		**x**					4	29
	Dyskinesia											**x**				1	7
	Pain				**x**	**x**					**x**	**x**				4	29
	Tiredness	**x**				**x**		**x**			**x**		**x**	**x**		6	43
	Difficulty performing activities of daily living		**x**			**x**			**x**	**x**	**x**	**x**	**x**	**x**		8	57
	Decreased functional independence		**x**		**x**	**x**			**x**							4	29
	**Gait changes**		**x**	**x**				**x**	**x**		**x**		**x**	**x**		**7**	**50**
	Fine motor difficulties		**x**			**x**							**x**			3	21
	**Weakness**		**x**					**x**	**x**	**x**	**x**	**x**	**x**	**x**		**7**	**50**
	Changes in the digestive system						**x**	**x**								2	14
	Postural Changes					**x**						**x**		**x**		3	21
	Loss of spontaneity												**x**			1	7
	Freezing of Gait												**x**			1	7
	Fear of falling	**x**	**x**		**x**	**x**					**x**			**x**		6	43
	Falls		**x**		**x**	**x**			**x**				**x**	**x**		6	43
	**Decrease in physical well-being**		**x**		**x**	**x**		**x**	**x**	**x**	**x**		**x**	**x**		**9**	**64**
Mental and emotional	Loss of Memory		**x**						**x**	**x**		**x**			**x**	5	36
	Feeling of incomprehension								**x**							1	7
	Distress and Despair		**x**										**x**			2	14
	Feeling of helplessness	**x**	**x**			**x**										3	21
	Shame		**x**	**x**									**x**			3	21
	Indisposition			**x**		**x**										2	14
	Changes to the voice		**x**	**x**					**x**						**x**	4	29
	Difficulty expressing yourself			**x**					**x**						**x**	3	21
	Sadness		**x**	**x**		**x**										3	21
	**Lower self-esteem**		**x**	**x**				**x**			**x**	**x**	**x**		**x**	**7**	**50**
	Less empowerment		**x**					**x**					**x**			3	21
	Impotence		**x**					**x**					**x**		**x**	4	29
	Sleep disorders					**x**				**x**	**x**		**x**			4	29
	**Concerns about the future**	**x**	**x**		**x**					**x**		**x**	**x**		**x**	**7**	**50**
	**Decreased emotional well-being**		**x**		**x**	**x**	**x**	**x**	**x**	**x**	**x**		**x**			**9**	**64**
	Feeling of not being yourself		**x**									**x**				2	14
Social	Fear to go out				**x**	**x**			**x**							3	21
	Lack of social interaction			**x**		**x**			**x**	**x**					**x**	5	36
	Inability to work		**x**			**x**							**x**			3	21
	Feeling people are staring at me			**x**			**x**				**x**	**x**	**x**			5	36

The bold values emphasize the responses with higher percentages.

### 3.2 Self-perceived QoL

When asked about their understanding of the meaning of QoL, some participants reported that QoL changes throughout the stages of life and according to the context in which the person is situated: *“It depends a lot on the phase of life that you are in. As a child, it’s one type, in adolescence, it’s another, in adulthood, it’s a different again. In mature life too, it’s different” (Violet).*

Considering the participants’ social-economic contexts, some emphasized the importance of the economic conditions, access to resources, and social support, especially from family and friends: *“Quality of life is also subjective. It depends on age, social conditions, and educational background… My experience with Parkinson’s tells me the understanding and support of my family and all my friends are indispensable…” (Violet); “It’s having someone who cares about you…” (Turquoise).*

Many participants highlighted health, the ‘will to live,’ and happiness as important factors in their QoL: *“It’s having health and the disposition to face the challenges that life presents” (Violet); “It’s about having good health, feeling pleasure” (Yellow); “I think it’s about being happy, being able to smile” (Turquoise); “It’s having health, experiencing pleasure in life, and being happy… I believe it’s crucial for us to be happy and have the will to live” (Red)*.

Independence and the freedom to do what they want were also aspects present in many statements: “*For me, it means having maximum independence in my life, minimal physical and emotional limitations, and the ability to do things… to be physically well” (Mustard); “Being able to do the things that you want to do…” (Brown); “For me, it’s about being able to go out, attend church, work, and be independent… That, to me, is quality of life. Not staying idle and wasting time” (Dark Green); “It’s about being able to do the things you enjoy, even if it’s at a slower pace” (Navy Blue).*

**Figure 3:**
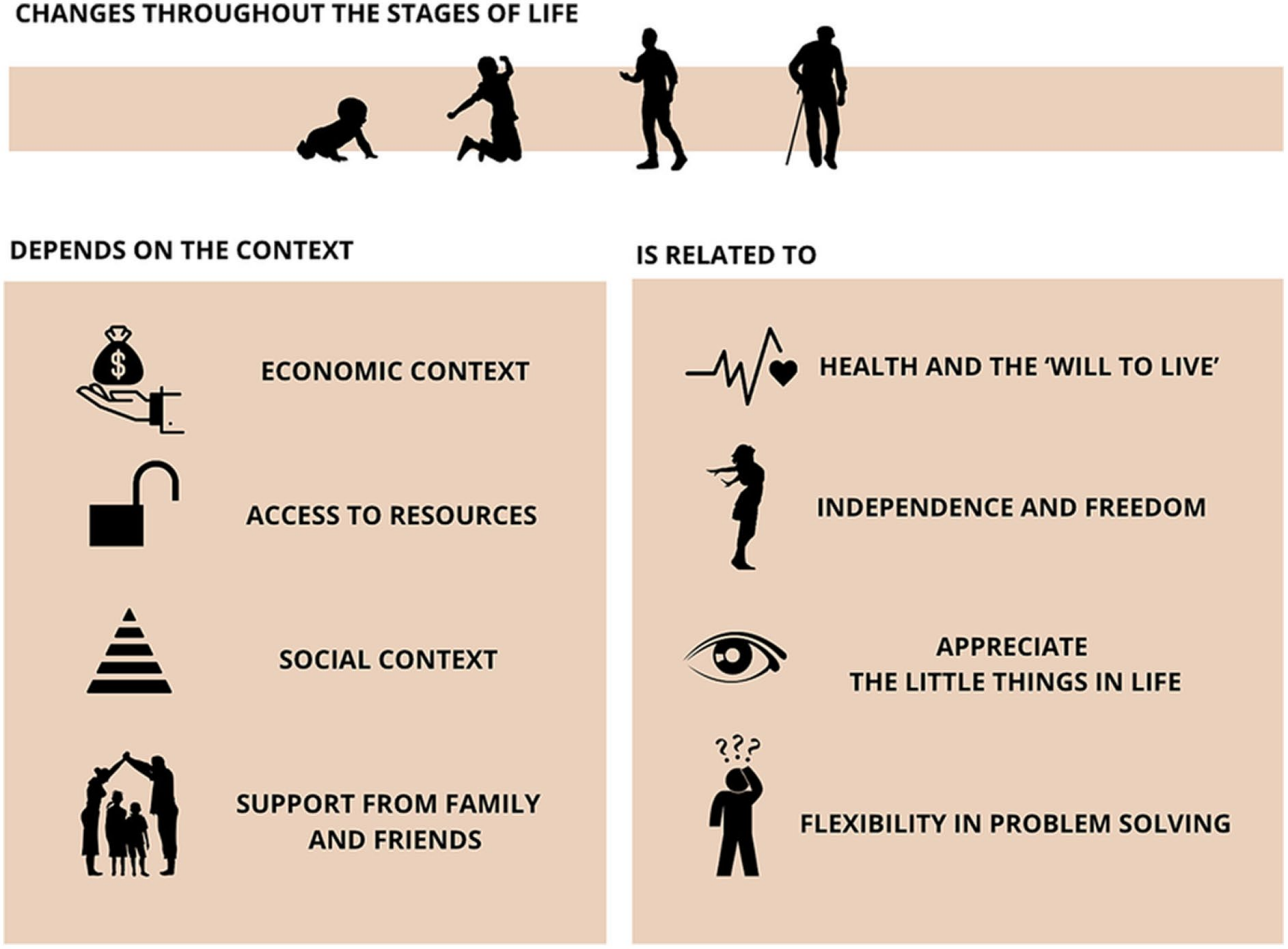
Self-perceived concept of QoL.

Considering the chronic nature of the disease and the wisdom acquired throughout life, some participants reflected on the importance of actively seeking QoL, valuing the little things in life, and being flexible in dealing with problems: *“It’s about trying to make each day a good day, choosing it, and striving to make it happen” (Lilac); “Being able to participate in things in the best way you can…” (Navy Blue); “It’s about having tranquillity, knowing how to appreciate the good times and having the balance to face and resolve the not-so-good ones. All of these contribute to our quality of life, I believe” (Violet).* The Figure 3 illustrates the self-perceived concept of QoL.

### 3.3 Self-perceived impacts of the in-person Brazilian dance classes

The participants highlighted the significance of their involvement in the “Dance & Parkinson” project in their lives: *“This project improves the quality of life for us a lot” (Red); “The classes are enjoyable!” (Yellow); “I felt much, much, much better being part of the Dance & Parkinson’s project” (Violet); “And it’s a place where you meet people with the same problem as you, and everybody smiles, everybody plays and dances.” (Turquoise); “I wanted to shout to the world: Everyone who has Parkinson’s should participate in this project, you know?” (Lilac).*

They shared their thoughts on the dance experience*: “Dance taught me a lot of things, still teaches me, to move, an arm this way, a leg that goes that way, and we do it” (Burgundy); “It’s the rhythm, it’s the movement, transforming the sound of the music into a body movement.” (Mustard);* They also reflected on the role of dance in humanity*: “I think that dance has a special magic […] I think dancing is fundamental. […] I think that if everyone in the world danced, the world would be a much better place” (Turquoise).*

The participants mentioned various positive physical, mental, emotional, and social impacts of the “Dance & Parkinson” project: *“Without a doubt, the dance classes are contributing in all aspects, physical and social, through gestures, affection, words. In physical and emotional well-being,… just getting a good morning like this with a smile, I’ve already won my day” (Lilac); “I feel much better. I do not know… the classes take away all that sickness thinking of things like that… I feel good!” (Red); “I feel the influence of the dance classes in social life, in daily life […] Besides the physical part, obviously, the mobility, the agility, the balance” (Violet).* All impacts reported by the participants were summarized in Table 4.

**Table 4:** The self-perceived impacts of the in-person Brazilian dance classes.

Subcategories of the impacts of Brazilaian dance classes		Lilac	Turquoise	Yellow	Orange	Red	Navy Blue	Mustard	Light Green	Brown	Violet	Burgundy	Dark Green	Pink	Gray	n	%
Physical	**Reduced stiffiness**	**x**	**x**		**x**			**x**			**x**	**x**		**x**		**7**	**50**
	Feeling of lightness and fluidity in the movements		**x**				**x**	**x**		**x**		**x**				5	36
	Agility		**x**			**x**					**x**			**x**		4	29
	Less rigidity		**x**					**x**			**x**	**x**		**x**		5	36
	Less pain		**x**			**x**										2	14
	**Improved balance**	**x**			**x**			**x**			**x**	**x**			**x**	**6**	**43**
	Disposition	**x**	**x**			**x**		**x**			**x**					5	36
	**Easier execution of activities of daily living**		**x**	**x**		**x**					**x**	**x**	**x**			**6**	**43**
	Improved motor coordination							**x**						**x**		2	14
	Improved stamina										**x**					1	7
	Easier to initiate movement		**x**					**x**		**x**						3	21
	Improved fine motor skills		**x**										**x**			2	14
	Improved body awareness											**x**				1	7
	Improved gait											**x**	**x**	**x**	**x**	4	29
	**Improved physical well-being**	**x**	**x**		**x**	**x**		**x**	**x**	**x**	**x**	**x**	**x**		**x**	**11**	**79**
Mental and emotional	**Happiness**	**x**	**x**		**x**		**x**				**x**	**x**	**x**		**x**	**8**	**57**
	**Enjoyment**		**x**		**x**		**x**	**x**			**x**	**x**	**x**		**x**	**8**	**57**
	Improved mood and liveliness	**x**	**x**				**x**	**x**				**x**		**x**		6	43
	Increased desire and motivation		**x**		**x**			**x**			**x**	**x**	**x**			6	43
	Increased energy and disposition	**x**	**x**			**x**		**x**			**x**	**x**				6	43
	**Feeling of pleasure**		**x**	**x**	**x**		**x**	**x**			**x**	**x**				**7**	**50**
	Feeling of freedom		**x**													1	7
	Feeling of lightness						**x**	**x**	**x**				**x**			4	29
	Fewer thoughts about illness					**x**			**x**							2	14
	Better able to face the difficulties of the disease		**x**					**x**		**x**		**x**				4	29
	Exercising memory	**x**				**x**	**x**									3	21
	Improved self-esteem	**x**	**x**		**x**	**x**	**x**						**x**			6	43
	Feeling of empowerment	**x**	**x**			**x**	**x**						**x**	**x**		6	43
	Feeling of self-confidence		**x**				**x**						**x**			3	21
	**Improved emotional well-being**	**x**	**x**		**x**	**x**	**x**	**x**	**x**	**x**	**x**	**x**	**x**		**x**	**12**	**86**
Social impacts	**Friendship**	**x**	**x**		**x**	**x**	**x**	**x**	**x**		**x**	**x**	**x**	**x**	**x**	**12**	**86**
	A greater sense of living										**x**		**x**	**x**		3	21
	**Increased participation in social activities**	**x**	**x**	**x**		**x**		**x**	**x**	**x**	**x**	**x**	**x**	**x**	**x**	**12**	**86**
	Increased communication												**x**			1	7
	Participating in dance performances	**x**				**x**						**x**				3	21
	Possibility of returning to work												**x**			1	7
	**Feeling of belonging**	**x**	**x**	**x**			**x**	**x**	**x**	**x**	**x**	**x**	**x**	**x**		**11**	**79**
	Possibility expressing yourself		**x**	**x**		**x**			**x**			**x**	**x**			6	43
	**Possibility to interact with peers**	**x**	**x**	**x**	**x**	**x**	**x**	**x**	**x**	**x**	**x**	**x**	**x**	**x**		**13**	**93**
	Feeling the support of the group		**x**						**x**			**x**	**x**			4	29
	Feeling of comprehension in the group			**x**		**x**			**x**			**x**	**x**			5	36

The bold values emphasize the responses with higher percentages.

Furthermore, all the participants underscored the importance of socialization and the sense of belonging provided by the group: *“The group brings vibrancy, joy, more energy…” (Lilac).* They emphasized the friendships they found: *“When we met and talked, it was so good! That’s why I liked it! We made other friends, we talked… even about the disease…” (Red);* The coexistence with the group motivates them to make more and more effort: *“This thing of the group, the motivation, of ‘go, go, go’ was the most important thing for me” (Dark Green); “The participation in this group gave me a certain encouragement… Not to let the disease take over … To fight against it…” (Mustard).*

In this context, the participants highlighted the positive aspect of finding a group of peers, where they felt free to talk and exchange experiences openly: *“Noticing people with the same problem as mine, sometimes worse than me, that helped me a lot to live with my disease” (Dark Green); “They are all the same. Everyone has difficulties, they understand everything…” (Yellow); “We speak the same language there … You feel good there… I think there is a release…” (Burgundy).* This coexistence with peers helps lessen the feeling of loneliness: *“We see that in the group we are not alone, you know? There are many people with the same problem. So there we get comfort” (Lilac); “There we feel inserted in the world and not isolated” (Violet); “The interaction with the teachers and with the other participants I think is very important because before it was me with Parkinson’s … I felt alone. And this interaction allowed us to realize we are not alone. And we feel part of the group too” (Turquoise).*

Many participants perceived an immediate positive effect during and after the in-person Brazilian dance class, reporting that they left the class better than they had arrived: *“I feel that whenever I left the class there, I felt much better than when I arrived… I came out with a lot of energy, a lot of disposition” (Mustard); “The state of mind improves, and the physical condition improves, and I feel more… I do not know if it’s the muscles that are more warmed up, stretched, I do not know what works, but it improves, I know it does!” (Turquoise); “The classes were good for my body and soul” (Violet).*

They perceived physical improvements: *“I started to feel more energy to do things. The energy is much better. And things start moving again!” (Turquoise); “The sensation we have after we finish the classes seems like you feel relieved, lighter…” (Navy Blue); “We had fun! I came out of there light!” (Dark Green).*

In addition to the immediate effects, participants perceived improvements over the years they have been participating in the project. Some pointed out physical impacts: *“I feel more agile. I think that dance helps me to be well… I can continue walking…” (Pink); “I went back to work, I went out again, talked more. I think it made me feel more at ease, right? And with the hope of getting better and better. So I felt much more confident because of that” (Dark Green).* Also, mental and emotional improvements were noted: *“I started attending, I started to have more confidence in myself… It increased more my self-confidence, the self-esteem” (Dark Green); “My clothes were always black or little brown… Suddenly, I started to arrive in colourful clothes, and that was drawing attention” (Lilac).*

The possibility of participating in some activities promoted the perception of empowerment and increased the self-esteem of some participants: *“I had never gone on stage to dance, right? And in the project, we even went on stage to dance. Everyone gave us a standing ovation… We prepared ourselves, we bought fabric, we made skirts, we dressed up, you know? Beautiful ones, you know? And that lifts our ego…” (Lilac); “We think we will not be able to do anything, but we dance things that we did not even think of dancing, do movements that we thought we would not be able to do!… We still can!… The dance class and the interaction, what we can do physically, improves our state of mind a lot; it gives us personal empowerment” (Turquoise).*

Another important aspect highlighted by the participants was the engagement and attendance in classes: *“The project was something that I always did with pleasure, right? So I think in the years I participated there, I do not remember missing a single day” (Mustard); “It’s hard for me to leave early in the morning, take the bus, in the cold, in the rain, but we never stop going” (Burgundy).* Some participants mentioned their motivation and preparation for class: *“The joy every day that we have class is very great; we get up with much more willpower” (Violet); “It was the best. I could not wait for the classes to happen because we would meet, wait for one or the other, you would arrive happy and leave even happier” (Navy Blue)*.

### 3.4 Self-perceived impacts of the online Brazilian dance classes

The COVID-19 pandemic and the social isolation experienced by the participants disrupted their lives, paused their activities, and changed their routines: *“It shook me when we had to stay still, it shook my head and my body, because we were just inside the house.” (Red); “Yeah, 24 h inside the house! You wake up and have breakfast, sit down and read the newspaper, watch TV, then do not have anything else to do. Then you become very idle, you cannot go out, you know, it’s dangerous…” (Gray).*

In some reports, the participants lamented the impossibility of having in-person dance classes in the “Dance & Parkinson” project: *“I am very sad the pandemic happened, that we had this isolation and did not continue the classes in-person…” (Violet); “We danced face-to-face and suddenly we had to stop that, and of course, that caused frustration…” (Mustard); “I miss the colleagues, now in the pandemic.” (Yellow); “It was an injustice to stop it, it was very good for me” (Gray).* And they perceived their PD symptoms worsened during social isolation: *“Until a few months ago, I was doing OK, and I was expressing myself well and I danced and did everything, went to the supermarket and now my symptoms are getting worse. […]” (Gray).*

About the online Brazilian dance classes during the pandemic, all the participants reported that they preferred in person classes, but were happy with the possibility of continuing to dance during social isolation: *“in person you vibrate more, your body corresponds better, right!” (Brown); “But I think it’s better this way [online] than standing still.” (Yellow); “It’s different, but it’s better than doing nothing.” (Navy Blue).* The participants mainly point to the lack of physical presence: *“[…] it is not the same interaction as when we are live and in colour, there the interaction is much greater. […] The human being feels the relationship, it does not matter, we feel the relationship” (Turquoise).* And several participants refer to the challenges faced in the online dance: *“Now, for example, I’m at home watching them, or only watching the teacher from afar, in a little while, for example, the internet fails, the voice disappears, there is not the same motivation or quality for us” (Navy Blue).*

Despite the limitations and challenges, they reported some results: *“I feel that online classes are limited, but the same, it has its results, not so good than in person, but it has results.” (Lilac); “It is quite different from the face-to-face project… but I will tell you, I spent a few days without dancing because I was injured, and I really missed it, I think it helps a lot so that we do not feel so far away from something that is so good for us, right?” (Orange).*

During the pandemic, the participants reported how online dance classes were part of their routine: *“I keep waiting for the class! I keep looking forward to it.” (Turquoise); “on Monday and Wednesday, we have the appointment. I think that’s important. […] The class is an incentive.” (Dark Green); “We get ready at night to get up earlier to get organized for class […] We do it because we like it, because we want to participate, we adapt and want to be there, and we are on time, to make the internet work.” (Navy Blue).*

They also reported positive effects perceived during and after the online dance classes: *“I feel very good after taking the virtual classes! […] I’ll tidy up, wash the clothes I wore, and I’ll put on some other clothes” (Red).* Even though it’s only on a screen, the participants expressed the importance of seeing their peers in the synchronous classes: *“It’s nice to see colleagues […] Online classes are very good, it’s the way we can see, at least from a distance, all the teachers, all the students.” (Violet); “When I can talk with someone, then I’m laughing by myself, you know? It always cheers me up…” (Lilac); “Given the longing we have for all the people, seeing each other on the screen improves us a lot.” (Yellow); “To see if there is anything new, if everyone is here, to see each one’s smile, what the teacher has to say, what the topic of the day is…” (Navy Blue).*

Table 5 summarizes the physical, mental, and emotional, and social self-perceived impacts of the online Brazilian dance classes during the COVID-19 pandemic.

**Table 5:** The self-perceived impacts of the online dance classes.

Subcategories of the impacts of online dance classes		Lilac	Turquoise	Yellow	Orange	Red	Navy Blue	Mustard	Light Green	Brown	Violet	Burgundy	Dark Green	Pink	Gray	n	%
Physical	Reduced stiffiness	**x**	**x**				**x**			**x**	**x**					5	36
	Agility	**x**									**x**			**x**		3	21
	Less Rigidity		**x**								**x**	**x**				3	21
	Less pain		**x**													1	7
	Improved balance										**x**	**x**				2	14
	Disposition		**x**				**x**				**x**				**x**	4	29
	Easier execution of activities of daily living		**x**	**x**		**x**						**x**			**x**	5	36
	Improved motor coordination		**x**									**x**				2	14
	Improved gait		**x**													1	7
	**Improved physical well-being**	**x**	**x**	**x**	**x**				**x**	**x**	**x**	**x**	**x**	**x**		**10**	**71**
Mental and emotional	Happiness		**x**			**x**	**x**				**x**	**x**				4	29
	Improved mood and liveliness	**x**	**x**				**x**					**x**				4	29
	Increased desire and motivation		**x**		**x**		**x**						**x**	**x**	**x**	6	43
	Increased energy and disposition		**x**		**x**	**x**	**x**				**x**					5	36
	Feeling of pleasure		**x**	**x**				**x**				**x**				4	29
	Feeling of lightness						**x**									1	7
	Feeling safe		**x**								**x**					2	14
	Better able to face the difficulties of the disease and pandemic				**x**		**x**							**x**		3	21
	Enhanced memory	**x**					**x**					**x**				3	21
	Intellectual stimulus	**x**														1	7
	Improved self-esteem		**x**		**x**	**x**	**x**						**x**	**x**		6	43
	Feeling of empowerment	**x**	**x**										**x**			3	21
	**Improved emotional well-being**	**x**		**x**	**x**		**x**		**x**		**x**	**x**	**x**			**8**	**57**
Social	**Friendship**	**x**	**x**		**x**		**x**	**x**			**x**	**x**	**x**	**x**		**9**	**64**
	Feeling of belonging		**x**	**x**				**x**								3	21
	Possibility expressing yourself			**x**												1	7
	**Possibility to see and interact with peers**	**x**	**x**	**x**	**x**	**x**	**x**	**x**	**x**		**x**		**x**	**x**		**11**	**79**
	Relieve the loneliness			**x**	**x**		**x**				**x**					4	29

The bold values emphasize the responses with higher percentages.

## 4 Discussion

The purpose of this study was to analyze the self-perceived impacts of Brazilian Dance on the QoL (physical, mental, emotional, and social aspects) of PwPD, both before and during the COVID-19 pandemic. The participants reported a wide range of factors that impact their QoL, depending on their circumstances and life stage (Figure 2). They also asserted that dancing had positive impacts on physical, mental, emotional, and social aspects of their QoL, both before and during the COVID-19 pandemic.

PD is a multifactorial condition that can affect each person differently (Poewe et al., 2017). Therefore, it is important to understand how PwPD self-perceived the impacts of PD in their QoL. The participants perceived several negative physical, mental, emotional, and social impacts in their lives following their Parkinson’s diagnosis. These findings align with existing literature, as PwPD often experience a range of motor and non-motor symptoms that affect their QoL (Poewe et al., 2017; Titova and Chaudhuri, 2018; Balestrino and Schapira, 2020). Regarding the physical aspects that negatively affect QoL, most participants mentioned rigidity, decrease in physical well-being, difficulty performing activities of daily living, among others. Regarding the mental and emotional impacts of PD on participants’ QoL, the primary issues reported included decreased emotional well-being, lower self-esteem, and concerns about the future. These perceptions are in line with the literature (Kuhlman et al., 2019; Verity et al., 2020; Kavya et al., 2022). These impacts can led to a decline in professional and social activities, potentially leading to a withdrawal from their usual social roles (Perepezko et al., 2019).

The COVID-19 pandemic has had a profound impact on the routine of PwPD (Brown et al., 2020; Subramanian et al., 2020). The reduction in participation in physical and social activities, the cancelation of medical appointments, and social isolation have contributed to the worsening of PD severity and the intensification of both motor and non-motor symptoms, consequently reducing the QoL for PwPD (Brown et al., 2020; Haas et al., 2022; Moratelli et al., 2022). The participant’s narratives emphasizing their ability to adapt to a new routine during the social isolation is align with the existing literature and underscore the detrimental impact of this abrupt change. In this context, engaging in online activities emerged as a crucial tool (Shalash et al., 2021), highlighting the significance of continuing online dance classes during the ongoing pandemic period (Delabary et al., 2022).

In terms of living with Parkinson’s, the participants expressed a view consistent with existing literature that highlights the negative impact of self-perceived QoL among PwPD (Stocchi et al., 2014). The presence of both motor and non-motor symptoms is known to influence this perception negatively (Bock et al., 2022). The participants detailed various aspects of living with the condition and underscored the challenges associated with diagnosis, the necessary adjustments in daily life, and the feeling of no longer being one’s true self. These findings align with Valcarenghi et al. (2018), who emphasized that living with a chronic illness can profoundly affect one’s life in numerous ways (Figure 3).

Given the chronic nature of PD and the wisdom that comes with life experiences, some participants reflected on the significance of resilience and acceptance in their pursuit of a higher QoL. They emphasized the need to adapt to their new reality, overcome challenges, and find joy in life’s smaller moments. Although resilience cannot alter the severity of PD, it has been associated with increased optimism and an improved QoL (Gardenhire et al., 2019). It also correlates with reduced disability, apathy, depression, and fatigue (Robottom et al., 2012). Acceptance and adaptability have an important role in perceived life satisfaction, especially in the context of a chronic and progressive condition like PD, where individuals must continuously adapt to new challenges (Rosengren et al., 2021).

Aligned with the World Health Organization’s definition (WHOQOL Group, 1993), participants perceived QoL as a subjective, individual, and multidimensional concept, impacted by physical, mental, emotional, and social factors. This perspective accounts for the experiences and perceptions of the person who grappling with the disease daily (Martinez-Martin, 2017). Considering this definition, Brazilian dance, both in-person and online, can be said to contribute toward improving physical, mental, emotional, and social aspects, and thus enhancing QoL for PwPD. Among the physical benefits of the in-person Brazilian dance classes perceived by the participants are improved physical well-being, reduced stiffness, improved balance, and eased execution of activities of daily living. Improvements in emotional well-being, happiness, enjoyment, and felling of pleasure were also emphasized as essential aspects of the in-person Brazilian dance classes. Also improvements in mood, self-esteem, feeling of empowerment and self-confidence were perceived, which are crucial for QoL in PwPD (García et al., 2019; Palmeri et al., 2019). These results corroborate with previous studies (Carapellotti et al., 2020; Emmanouilidis et al., 2021; Ismail et al., 2021; Zhou et al., 2021; Delabary et al., 2022; Feenstra et al., 2022). However, in addition, the participants reported experiencing other aspects less addressed in the literature, among them feeling of lightness and fluidity in movements, being able to perform daily life activities more easily, and increased agility and energy, all of which contribute to maintain functionality and QoL. Thus, dance can be considered an accessible, engaging, motivating, and enjoyable artistic expression that facilitates PwPD participation (McNeely et al., 2015; Emmanouilidis et al., 2021).

Regarding the online Brazilian dance classes, the participants reported a sense of growing confidence as they overcame technological barriers and engaged in the activities. They emphasized a sense of liveliness, desire and motivation, and energy and disposition. Other positive impacts on emotional and mental well-being were perceived, such as, enhanced memory, and greater strength to cope with the challenges posed by the disease and the pandemic. The literature on the effects of online dance classes in PwPD is relatively recent and has highlighted the benefits and challenges faced during the pandemic (Bek et al., 2021; Delabary et al., 2022). Among the main barriers are difficulty connecting with the internet, a lack of technological knowledge and decreased motivation to participate in dance activities (Bek et al., 2021; Delabary et al., 2022). Despite facing these challenges and missing the physical presence of their peers, the participants perceived several positive aspects, such as: the possibility to stay active by dancing at home, enhanced balance and posture, and improved mood, confidence, and motivation (Bek et al., 2021; Delabary et al., 2022).

While the participants perceived the above physical, mental, and emotional benefits of both in-person and online Brazilian dance classes, the social aspects received greater emphasis. However, the participants perceived greater social benefits with the presence of their peers in the in-person classes. Nevertheless, they stressed the importance of the online dance classes in alleviating feelings of isolation and permitting the interaction with peers during the pandemic. In addition to increased social participation and friendships, the in-person and online Brazilian dance classes fostered a sense of belonging within the group. The participants felt comfortable expressing themselves, engaging in discussions with peers about shared concerns, and receiving support and understanding. This social support was instrumental in reducing self-stigma, enhancing participation, and fostering self-expression. These perceived social benefits align with previous research (Earhart, 2009; Bognar et al., 2017) which demonstrated that dance can improve social participation and change perspectives and attitudes toward Parkinson’s.

Overall, the in-person and online Brazilian dance classes offers a nuanced understanding of their impacts on the multifaceted aspects of QoL for PwPD. The in-person Brazilian dance classes were associated with physical improvements, emotional well-being, and increased social benefits, while online classes were noted for overcoming technological barriers, maintaining activity levels, and supporting emotional and mental well-being during the pandemic. Socially, in-person classes provided a stronger sense of camaraderie, while online classes addressed isolation concerns. Both settings fostered a sense of belonging, social support, and improved social participation.

### Limitations

This study has some limitations which should be considered. Due the COVID-19 pandemic, the interviews had to be conducted virtually. Consequently, internet failures may have hampered communication. Additionally, in some cases, the presence of a family member or caregiver at home may have impacted the participants’ responses. Also, the study faced challenges due to the unprecedented circumstances of the pandemic, making it difficult to conduct comprehensive assessments typically performed in a clinical setting, such as the Unified Parkinson’s Disease Rating Scale (UPDRS), due to not conduct in-person interactions with the participants during this period.

## Conclusion

The participants perceived various challenges associated with living with Parkinson’s, all of which have a negative impact on their QoL. However, a recurring theme in their narratives is the significance of resilience, acceptance, and commitment to treatment in the process of overcoming these challenges and adapting to their new reality. The Dance & Parkinson’s project, whether conducted in-person or online (both before and during the COVID-19 pandemic), was shown to improve physical, mental, emotional, and social dimensions, thereby enhancing the participants QoL. The study underscores the potential relevance of Brazilian Dance interventions, including online approaches during the COVID-19 pandemic, as a valuable component in healthcare strategies for populations with chronic conditions.

## Data availability statement

The raw data supporting the conclusions of this article will be made available by the authors, without undue reservation.

## Ethics statement

This study was approved by the Federal University of Rio Grande do Sul Ethics Committee (CAAE 190 33547920.9.0000.5347). The studies were conducted in accordance with the local legislation and institutional requirements. The participants provided their written informed consent to participate in this study.

## Author contributions

MD: Conceptualization, Data curation, Formal analysis, Investigation, Methodology, Project administration, Writing – original draft, Writing – review & editing. IL: Data curation, Formal analysis, Methodology, Writing – original draft, Writing – review & editing. ET: Data curation, Methodology, Writing – original draft, Writing – review & editing. CG: Data curation, Formal analysis, Methodology, Writing – original draft, Writing – review & editing. AN: Conceptualization, Data curation, Formal analysis, Investigation, Methodology, Project administration, Supervision, Writing – original draft, Writing – review & editing.

## Footnotes

^1^https://www.youtube.com/channel/UCILPKVSA-7LygQ-zAh5wR3A
